# Hormetic Effects of Curcumin in RPE Cells: SIRT1 and Caspase-3 Inactivation with Implications for AMD

**DOI:** 10.3390/ijms26178555

**Published:** 2025-09-03

**Authors:** Jacopo Di Gregorio, Darin Zerti, Giulia Carozza, Annamaria Capozzo, Vincenzo Flati, Marco Feligioni, Rita Maccarone

**Affiliations:** 1 Department of Biotechnological and Applied Clinical Sciences, University of L’Aquila, 67100 L’Aquila, Italy; jacopo.digregorio@guest.univaq.it (J.D.G.); darin.zerti@univaq.it (D.Z.); giula.carozza@newcastle.ac.uk (G.C.); annamaria.capozzo@univaq.it (A.C.); vincenzo.flati@univaq.it (V.F.); 2 Fondazione European Brain Research Institute (EBRI) Rita Levi-Montalcini, 00161 Rome, Italy; m.feligioni@ebri.it; 3 Department of Neurorehabilitation Sciences, Casa di cura IGEA, 20144 Milan, Italy

**Keywords:** RPE, curcumin, oxidative stress, AMD, SIRT1, acetyl-p53, caspase-3

## Abstract

Retinal Pigment Epithelium (RPE), a component of the blood–retinal barrier, plays a pivotal role in maintaining retinal homeostasis and visual function. Dysfunction of the RPE is an early event that triggers photoreceptor death, in Age-related Macular Degeneration (AMD), a multifactorial disorder primarily caused by an imbalance between endogenous antioxidant defenses and reactive oxygen species production. Our in vitro study investigated the hormetic effects of curcumin in human RPE cells (ARPE-19), focusing on its capability to modulate two enzymes related to the onset of AMD: Sirtuin 1 (SIRT1), a NAD+-dependent deacetylase enzyme involved in cellular metabolism, aging, and stress response, and caspase-3, a crucial enzyme in programmed cell death. Curcumin exhibited classic hormetic doseresponses, with low concentrations (5–10 μM) providing cytoprotection while at high doses (≥20 μM) inducing toxicity. Under moderate oxidative stress, acetylated p53 was significantly reduced, indicating SIRT1 activation; curcumin 10 μM restored basal SIRT1 activity, while 5 µM did not. Both concentrations significantly decreased cleaved caspase-3 levels, demonstrating the anti-apoptotic effects of curcumin. Our results reveal curcumin’s hormetic mechanisms of RPE protection and emphasize the critical importance of dose optimization within the hormetic window for AMD therapeutic development.

## 1. Introduction

### 1.1. RPE Structure and Function

The Retinal Pigment Epithelium (RPE) is a monolayer of polarized, tightly connected, and pigmented cells located close to the neuroretina and forms the blood–retinal barrier together with the Bruch’s membrane and choriocapillaris [1,2]. The apical membrane of the RPE cells closely interacts with the photoreceptors’ outer segments. The RPE cooperates with photoreceptors in the visual cycle by recycling pigments of the visual cascade and plays a key role in degrading the outer segments of photoreceptors by phagocytosis [2,3,4]. Furthermore, the RPE transports nutrients and oxygen from the choriocapillaris to the photoreceptors [5], a process that requires high metabolic activity, increased oxygen consumption, and consequently leads to elevated production of reactive oxygen species (ROS) [2,6,7].

### 1.2. Oxidative Stress and AMD Pathogenesis

When an imbalance between ROS production and endogenous antioxidant machinery activation occurs, oxidative stress is triggered and RPE homeostasis is lost [5,8]. RPE dysfunction is one of the first events that occurs in several retinal disorders, including Age-related Macular Degeneration (AMD), the leading cause of irreversible blindness in developed countries [9,10]. AMD is a multifactorial disease in which oxidative stress plays a key role in the RPE dysfunction and photoreceptor loss [11].

### 1.3. SIRT1 in Cellular Homeostasis

The NAD+-dependent histone deacetylase Sirtuin 1 (SIRT1) is a crucial regulator of cellular responses to stress, including oxidative stress, and has been shown to play a significant role in AMD progression [12,13]. Histone deacetylases (HDACs) are important epigenetic regulators that deacetylate lysine residues on specific histone and non-histone proteins. Based on the homologies of their respective yeast orthologs, human HDACs are classified into four groups. The human sirtuin family, part of the class III HDACs, is a group of NAD+-dependent deacetylases and includes seven members (SIRT1-7) [14]. Sirtuins are involved in the regulation of several cellular processes such as metabolism, mitochondrial homeostasis, autophagy, DNA repair, redox balance, apoptosis, and senescence. Among the different sirtuins, SIRT1 is the most studied, responsible for regulating a wide range of biological processes, including cell cycle regulation, DNA repair, apoptosis, inflammation, autophagy, and aging [15,16]. SIRT1 is also involved in the pathogenesis of chronic conditions such as diabetes, pulmonary diseases, neurodegenerative diseases, and cardiovascular diseases. SIRT1 exerts its activity through the deacetylation of lysine residues on histone and non-histone proteins [16,17]. Among SIRT1 targets, p53 is one of the first non-histone substrates identified as functionally associated with the anti-senescence activity of SIRT1 [18]. SIRT1 inhibits p53 transcriptional activity by deacetylating the lysine residue at position 382 (K382), reducing p21 expression [15]. Hence, p21 is a transcriptional target of p53, and its expression inhibits cyclin-dependent kinase (CDK) activity and regulates cell cycle arrest during cellular senescence. Deacetylation of p53 by SIRT1 results in the inhibition of DNA damage and stress-mediated cellular senescence responses.

### 1.4. SIRT1 Dysregulation in AMD

SIRT1 dysregulation has been observed in both AMD patients and in cellular and animal models of the disease. Since RPE cell dysfunction is a major contributor to the development of AMD, many studies have focused on evaluating whether and how SIRT1 is involved in RPE cell dysfunction. AMD-derived RPE has been shown to exhibit increased expression of PARP2 (poly (ADP-ribose) polymerase 2), resulting in decreased NAD+ levels and a significant reduction in SIRT1 protein levels in the RPE of affected donors compared to normal RPE. This led to a dysfunctional AMPK/SIRT1/PGC-1α pathway [19]. Furthermore, another study has shown a correlation between decreased SIRT1/PGC-1α proteins and AMD pathogenesis in the RPE derived from iPSCs from an AMD donor [20]. This study indicated that dysfunctional SIRT1/PGC-1α could reduce mitochondrial activity and increase ROS production, contributing to the pathophysiology of AMD. SIRT1 modulation and function have also been studied in in vitro and in vivo models of AMD with contrasting results. Some studies have shown a decrease in SIRT1 expression following oxidative stress, while others suggest an increase [19,21,22,23,24,25,26]. Despite these discrepancies, there is consensus that SIRT1 is altered in AMD pathogenesis, highlighting its potential as a therapeutic target. Given the critical role of SIRT1 in maintaining cellular homeostasis, particularly under stress conditions, modulating its activity may offer therapeutic benefits for AMD.

### 1.5. Curcumin as a Therapeutic Agent

Curcumin is the main active component of the rhizome of *Curcuma longa*, first chemically characterized in 1910 as a low-molecular-weight polyphenol [27]. The therapeutic potential of curcumin has always been associated with its antioxidant properties, making it particularly effective in conditions where oxidative stress plays a significant role [28,29,30]. Moreover, in recent years, several studies have demonstrated the utility of curcumin in the prevention and treatment of inflammatory diseases. Notably, several studies have also revealed the ability of curcumin in modulating SIRT1-related pathways in non-ocular tissues, such as HUVECs and cardiomyocytes, as well as in an animal model of diabetes [17,31,32,33]. This modulation could represent a key mechanism through which curcumin exerts its protective effect on RPE cells. Our previous results have shown a dose-dependent effect on ARPE-19 cells when exposed to curcumin. Indeed, we demonstrated that higher concentrations of curcumin (0.05 mM and 0.1 mM) are able to decrease the RPE proliferation, while at lower concentrations (0.01 mM) it promotes RPE health and survival [34]. Furthermore, curcumin is well known to reduce the levels of nuclear factor kappa-light-chain-enhancer of activated B cell (NF-κB) and ROS [35] in experimental models of AMD, but to date, it has not been clarified whether curcumin targets single downstream events of the SIRT1-mediated enzymatic cascade, or whether its pleiotropic effect may be the result of the regulation of SIRT1 itself.

### 1.6. Hormesis and Curcumin: A Biphasic Dose–Response Relationship

The therapeutic effects of curcumin exhibit a characteristic biphasic dose–response pattern consistent with hormesis, a fundamental biological phenomenon where low doses of a substance produce beneficial effects while higher doses may be neutral or harmful [36,37]. Hormesis is defined as an adaptive response of cells and organisms to moderate stress, characterized by low-dose stimulation of protective mechanisms and high-dose inhibition or toxicity [38].

In the context of curcumin and cellular health benefits, this hormetic response has been observed across various cell types and experimental models. Low concentrations of curcumin typically activate cytoprotective pathways, including antioxidant enzyme upregulation, stress response protein expression, and cellular repair mechanisms. Conversely, higher concentrations may overwhelm cellular homeostatic mechanisms, leading to oxidative stress, mitochondrial dysfunction, and cell death [39,40].

The hormetic nature of curcumin is particularly relevant in RPE cell biology, where the balance between oxidative stress and antioxidant defenses is critical for cellular survival. Our previous findings demonstrated this biphasic response in ARPE-19 cells, where curcumin at 0.01 mM promoted cell health and survival, while concentrations of 0.05–0.1 mM decreased cell proliferation [34]. This hormetic effect may be mediated through the modulation of key cellular pathways, including SIRT1-dependent mechanisms, which regulate cellular stress responses, DNA repair, and metabolic homeostasis.

Understanding the hormetic properties of curcumin is essential for determining the optimal therapeutic windows and may explain the variable results observed in different experimental conditions. The identification of hormetic dose ranges for curcumin in RPE cells could provide valuable insights for developing effective therapeutic strategies for AMD, ensuring maximum protective benefits while avoiding potential adverse effects.

### 1.7. The Aim of This Study

The present study aimed to investigate the potential protective effects of curcumin on RPE cells exposed to oxidative stress, with a particular focus on its interaction with the SIRT1 pathway. The study utilized the ARPE-19 cell line under oxidative stress, induced by hydrogen peroxide (H_2_O_2_), to evaluate the impact of curcumin on cell viability, SIRT1 enzymatic activity, and its downstream molecular targets [17,18]. Among the latter, we investigated the activation of caspase-3, a key executioner protease in the apoptotic process. Oxidative stress triggers caspase-3 activation by proteolytic cleavage, contributing to RPE cell death, an early event in AMD pathogenesis [10,11].

Several studies have reported that curcumin, in addition to modulating SIRT1 activity, can attenuate caspase-3 activation in both cellular [41] and animal models of retinal neurodegenerative and inflammatory diseases, suggesting a potential anti-apoptotic effect [35]. Particularly, a recent study has shown that curcumin can mitigate oxidative stress and reduce apoptosis in ARPE-19 cells by modulating the SIRT1/Nrf2/HO-1 axis, as well as by downregulating cleaved caspase-3 expression [41]. These findings suggest that the protective effects of curcumin on RPE cells may involve both the upstream modulation of SIRT1, as well as the downstream inhibition of pro-apoptotic effectors such as caspase-3. Therefore, this study aimed to provide insight into the molecular mechanisms by which curcumin may exert a protective action on RPE cells by regulating SIRT1 activity together with the inhibition of caspase-3 activation, thus contributing to a better understanding of the molecular mechanisms underlying its therapeutic potential in AMD.

## 2. Results

### 2.1. Effects of Curcumin on ARPE-19 Cell Viability Under Oxidative Stress Conditions

**Figure 1:**
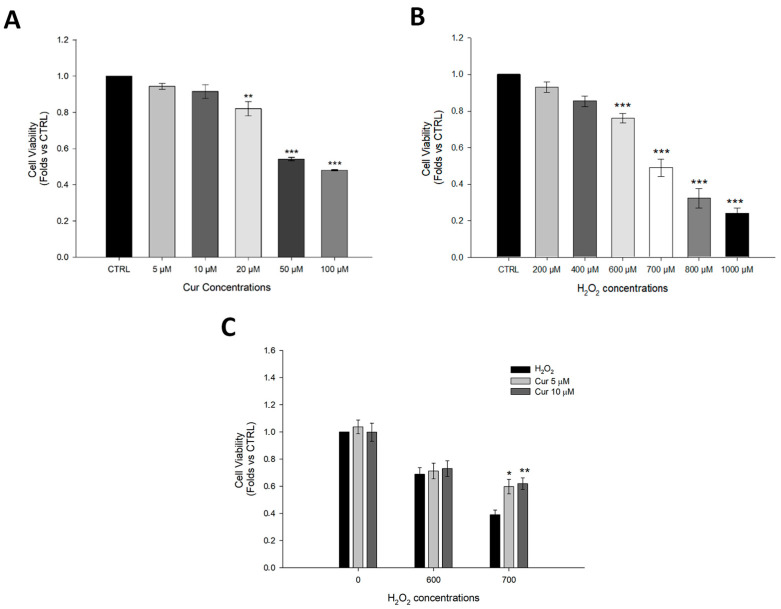
**Evaluation of curcumin toxicity and its protective effect against H_2_O_2_**. (**A**) MTT assay to evaluate cell viability in ARPE-19 cells treated for 24 h with curcumin at the following concentrations: 5 µM, 10 µM, 20 µM, 50 µM, and 100 µM. (**B**) MTT assay to evaluate cell viability in ARPE-19 cells treated for 24 h with H_2_O_2_ at the following concentrations: 200 µM, 400 µM, 600 µM, 700 µM, 800 µM, and 1000 µM. (**C**) MTT assay to evaluate cell viability in ARPE-19 cells pre-treated with curcumin (5 µM, 10 µM) for 24 h, followed by treatment with H_2_O_2_ (600 µM, 700 µM) for 24 h. Statistical analysis was performed using One-Way ANOVA followed by Holm–Sidak Test (n = 3–9). * *p* < 0.05; ** *p* < 0.01; *** *p* < 0.001. (**B**) Abbreviation: CTRL; control and Cur; curcumin. Data are shown as Mean ± SE.

The graph in Figure 1A shows that at concentrations from 20 to 100 µM of curcumin, cell viability significantly decreased; hence, for the next experiments, we selected the concentrations 5 µM and 10 µM. The plot in Figure 1B demonstrates that H_2_O_2_ from 600 up to 1000 µM was able to reduce the cell viability in a range between 80% and 30%; therefore, all the following experiments were performed with H_2_O_2_ at 600 and 700 µM to induce moderate damage. As shown in Figure 1C, 24 h pre-treatment with curcumin at 5 µM and 10 µM was able to significantly preserve cell viability by counteracting the oxidative stress induced by 700 µM H_2_O_2_.

### 2.2. Effects of the SIRT1 Activator SRT1720 and the Inhibitor EX527 on ARPE-19 Cell Viability

**Figure 2:**
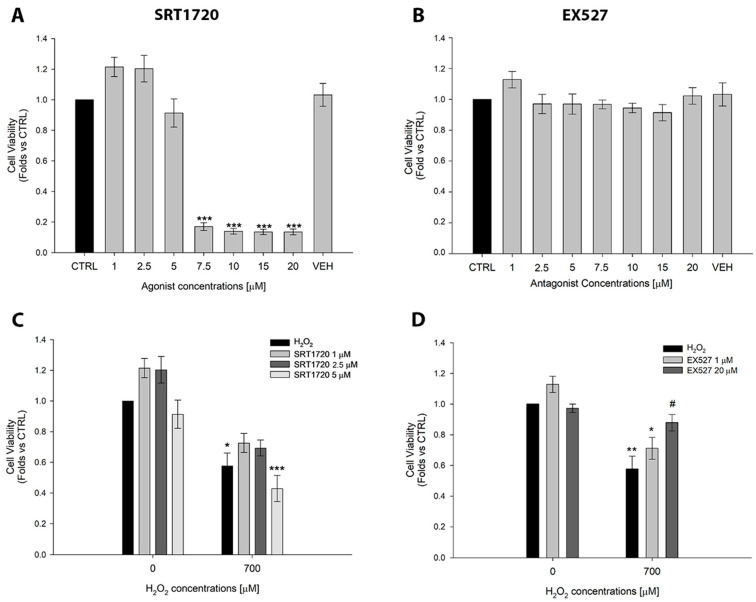
**Effects of the SIRT1 activator and inhibitor on ARPE-19 cells**. (**A**) Cell viability of ARPE-19 cells exposed to increasing concentrations of SIRT1 agonist SRT1720. (**B**) Cell viability of ARPE-19 exposed to increasing concentrations of SIRT1 inhibitor EX527. (**C**) Cell viability of ARPE-19 pre-treated with SRT1720 (1, 2.5, and 5 µM) and then exposed to H_2_O_2_ 700 µM for 24 h. (**D**) Cell viability of ARPE-19 pre-treated with EX527 (1 and 20 µM) and exposed to H_2_O_2_ 700 µM for 24 h. Abbreviation: CTRL; control; VEH; vehicle, and Cur; curcumin. Statistical analysis was performed using the One-Way ANOVA test followed by the Student–Newman–Keuls Method (n = 3). * *p* < 0.05; ** *p* < 0.01; *** *p* < 0.001 vs. CTRL; # *p* < 0.05 vs. H_2_O_2_. Data are shown as Mean ± SE.

Before investigating the curcumin effect on the SIRT1 pathway, a pilot experiment was performed to evaluate the cellular response to SIRT1 modulation (Figure 2A,B) in ARPE-19 cells by using the selective activator SRT1720 or the inhibitor EX527 [24]. We demonstrated that SRT1720 has a dose-dependent effect in reducing cell viability, as reported in Figure 2A. The concentrations of SRT1720 between 7.5 and 20 µM induced a significant loss of cells. These results suggested that the hyperactivation of SIRT1 is detrimental to cell survival, as previously shown [24]. All the concentrations of EX527 tested did not cause any significant change in cell viability, as shown in Figure 2B. Based on these results, we selected the non-cytotoxic concentrations of SRT1720 and EX527 to perform the next set of experiments under oxidative stress. We selected 1, 2.5, and 5 µM of SRT1720 and 1 and 20 µM of EX527, respectively. Our results demonstrated that the activation of SIRT1 with 1, 2.5, and 5 µM of SIRT1720 did not preserve the cell viability of ARPE-19 cells exposed to oxidative stress. Notably, the highest concentration of SRT1720 (5 µM) further reduced the cell viability after hydrogen peroxide treatment, corroborating that SIRT1 hyperactivation has a negative effect on cell viability (Figure 2C). Conversely, the highest concentration of SIRT1 inhibitor EX527 (20 µM) significantly improved cell viability in ARPE-19 cells under oxidative stress conditions, as reported in Figure 2D. These results suggest that the hyperactivation of SIRT1 is harmful for ARPE-19 cells, whereas its inhibition increases cell viability under oxidative stress conditions.

### 2.3. Curcumin Inhibits SIRT1 Activity and Protects Against SIRT1-Mediated Cell Death in ARPE-19 Cells

**Figure 3:**
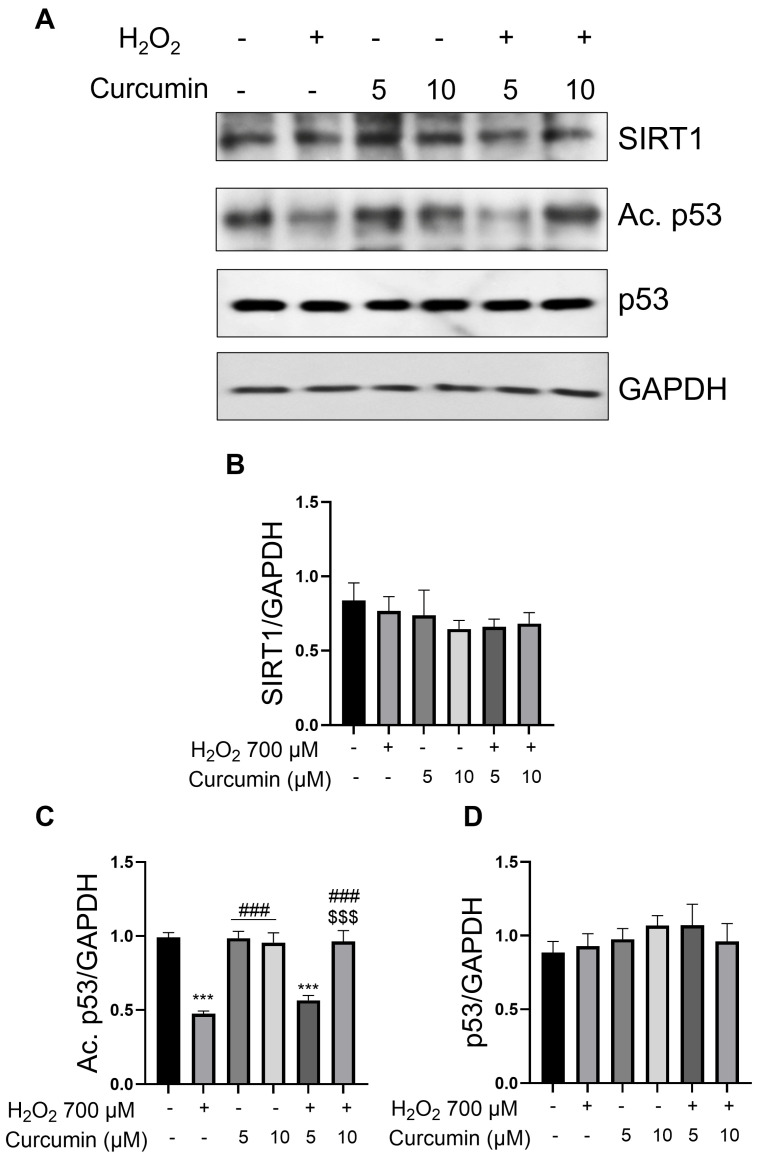
**Curcumin inhibits SIRT1 activity in ARPE-19 cells**. ARPE-19 cells were treated with 700 µM H_2_O_2_ and 5–10 µM of curcumin as indicated for 24 h. (**A**) Western Blot analysis of SIRT1, acetylated p53, and p53. (**B**–**D**) Densitometric analysis of the proteins from (**A**) normalized against GAPDH. Statistical analysis was performed using One-Way ANOVA followed by Tukey Test (n = 4). *** *p* < 0.001 vs. CTRL; ### *p* < 0.001 vs. H_2_O_2_; $$$ *p* < 0.001 vs. Cur5 + H_2_O_2_. Data are shown as Mean ± SE.

To analyze at the molecular level the effects of oxidative stress on SIRT1, as well as the protective effects of curcumin against SIRT1 activity in ARPE-19 cells, we first evaluated the SIRT1 protein levels in all experimental groups and no modulation of SIRT1 was induced by either H_2_O_2_ or curcumin (Figure 3A,B), indicating that functional changes in SIRT1 occur through activity modulation rather than alterations in protein expression levels. Given the unchanged SIRT1 protein levels, we investigated whether curcumin could modulate the acetylated form of p53, a well-characterized substrate of SIRT1 deacetylase activity [42]. The acetylation status of p53 serves as a reliable indicator of SIRT1 enzymatic activity, and in ARPE-19 cells, p53 deacetylation has been previously associated with apoptosis activation [43,44], making this target particularly relevant in the context of oxidative stress-induced RPE dysfunction. Interestingly, we observed a significant reduction in p53 acetylation following H_2_O_2_ treatment when compared to control cells (Figure 3A,C). This reduction indicates an enhanced SIRT1 deacetylase activity under oxidative stress conditions. Importantly, total p53 protein levels remained unchanged (Figure 3A,D), confirming that the observed changes reflect SIRT1-mediated deacetylation rather than alteration in substrate availability. Remarkably, only the treatment with curcumin at 10 µM and H_2_O_2_ 700 µM prevented the oxidative stress-induced p53 deacetylation (Figure 3A,C). This finding demonstrates the capability of 10 µM of curcumin to restore physiological SIRT1 deacetylase activity when it becomes dysregulated. Taken together, our data reveal a dose-dependent modulation of SIRT1 activity by curcumin that affects its downstream targets.

### 2.4. Curcumin Mitigates Oxidative Stress-Induced Apoptosis via Caspase-3 Modulation

**Figure 4:**
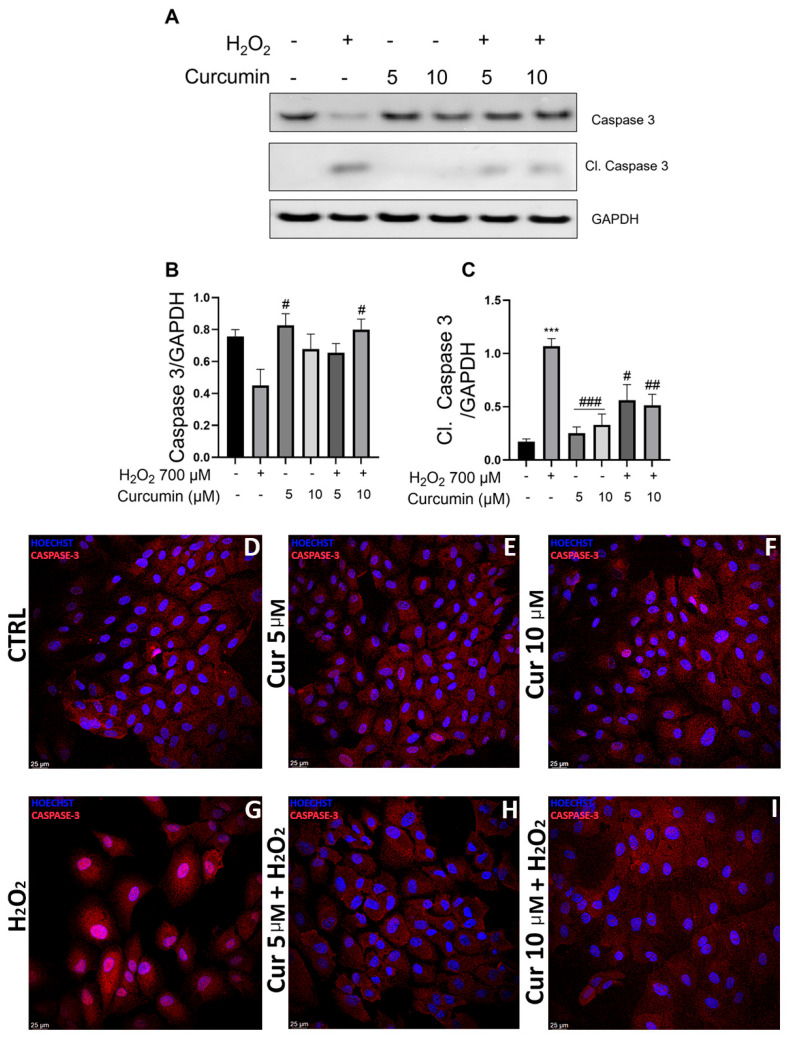
**Curcumin protects against cell death in ARPE-19 cells through caspase-3 modulation**. ARPE-19 cells were treated with 700 µM H_2_O_2_ and 5–10µM of curcumin as indicated for 24 h. (**A**) Western Blot analysis of caspase-3 and cleaved caspase-3. (**B**,**C**) Densitometric analysis of the proteins from (**A**) normalized against GAPDH. Caspase-3 staining of ARPE-19 cells: representative confocal images of ARPE-19 cells stained with caspase-3 (red) and bisbenzimide (blue) in (**D**) control cells; (**E**) cells treated with curcumin 5 µM; (**F**) cells treated with curcumin 10 µM; (**G**) cells exposed to 700 µM H_2_O_2_; (**H**) cells treated with curcumin 5 µM and exposed to 700 µM H_2_O_2_; (**I**) cells treated with curcumin 10 µM and exposed to 700 µM H_2_O_2_. Scale bar: 25 µm. Abbreviation: CTRL; control; CUR; curcumin. Statistical analysis was performed using One-Way ANOVA followed by Tukey Test (n = 4). *** *p* < 0.001 vs. CTRL; # *p* < 0.05, ## *p* < 0.01, ### *p* < 0.001 vs. H_2_O_2_. Data are shown as Mean ± SEM.

To evaluate the effects of SIRT1 modulation on apoptotic pathways, we assessed caspase-3 activation by evaluating its cleaved form, using the same protein extracts used for SIRT1 analysis, as shown in Figure 3. We measured both full caspase-3 and cleaved (activated) form levels (Figure 4A,C). Consistent with cell viability data (Figure 1), H_2_O_2_ treatment induced a significant activation of apoptosis, as evidenced by increased caspase-3 cleavage (Figure 4A,C). Interestingly, both curcumin concentrations (5 and 10 µM), when administered in combination with H_2_O_2_, significantly reduced cleaved caspase-3 levels, effectively protecting ARPE-19 cells against oxidative stress-induced cell death (Figure 4C). These results demonstrate that curcumin exerts anti-apoptotic effects through downregulation of caspase-3 activation in ARPE-19 cells under oxidative stress conditions.

To corroborate the molecular analysis and provide spatial resolution of caspase-3 activation, we performed immunofluorescence analysis to directly visualize how curcumin impacts its activation in ARPE-19 cells under oxidative stress. Although the antibody used for the analysis detects both full and cleaved caspase-3 forms, it shows distinct caspase-3 subcellular localizations: the cleaved (activated) form is detectable in the nucleus [45], indicating elevated apoptosis, while the inactive form exhibits cytoplasmic localization [46,47]. Immunofluorescence analysis revealed markedly higher nuclear expression of activated caspase-3 in the H_2_O_2_ group (Figure 4G), when compared to control (Figure 4D), with concurred strong cytoplasmatic immunoreactivity of the inactive form. This subcellular redistribution pattern is characteristic of apoptosis initiation and confirms the pro-apoptotic effects of oxidative stress observed in our biochemical analyses. Reduced nuclear expression of cleaved caspase-3 was observed in cells incubated with curcumin at both 5 and 10 µM concentrations, under basal conditions (Figure 4E,F) and under oxidative stress (Figure 4H,I). These findings demonstrate that curcumin prevents nuclear translocation of activated caspase-3, thereby inhibiting apoptosis. Confocal images also enabled the assessment of cellular morphology as an indicator of cellular health. In control (Figure 4D) and in curcumin 5 µM (Figure 4E) and 10 µM (Figure 4F) groups, the cells appeared in a confluent monolayer with tight cell–cell contacts indicative of a healthy status [48]. In contrast, H_2_O_2_ treatment resulted in loosely adherent, scattered cells with compromised morphology consistent with oxidative stress-induced cellular damage (Figure 4G). In accordance with the MTT assay (Figure 1C), these immunofluorescence data confirmed that H_2_O_2_ supplementation in ARPE-19 cells causes increased apoptosis, while the curcumin pretreatment preserves cell viability through caspase-3 inactivation (Figure 4). The convergence of biochemical markers (reduced level of cleaved caspase-3), subcellular localization changes (prevented nuclear translocation), and morphological preservation provides robust evidence for curcumin’s protective effects on ARPE-19 cells. Taken together, our results demonstrate that curcumin protects ARPE-19 cells from apoptosis under oxidative stress, through a coordinated mechanism involving SIRT1 activity modulation and caspase-3 activation, suggesting a mechanistic link between these pathways in RPE cell protection.

## 3. Discussion

The present study provides novel insights into the protective mechanisms of curcumin against oxidative stress-induced damage in Retinal Pigment Epithelium (RPE) cells, with particular emphasis on SIRT1 pathway modulation and apoptotic regulation. Our findings demonstrate that curcumin exerts dose-dependent neuroprotective effects in ARPE-19 cells through a dual mechanism: inhibition of SIRT1 hyperactivation and reduction of cleaved caspase-3-mediated apoptosis.

Our results confirm and extend previous findings regarding curcumin’s dose-dependent effects on RPE cells [34,49,50]. The protective concentrations of curcumin (5 and 10 μM) significantly preserved cell viability under oxidative stress conditions, while higher concentrations (≥20 μM) demonstrated cytotoxic effects. This biphasic response represents a classic example of hormesis, a well-documented phenomenon where low doses of a compound produce beneficial effects while higher doses become harmful [51]. The hormetic nature of curcumin’s effects has been extensively characterized across multiple cell types and experimental systems, consistently demonstrating that low doses have stronger beneficial effects than high doses [51]. Importantly, this dose-dependent dual functionality confers to curcumin a remarkable versatility: at low concentrations, it acts as an anti-apoptotic agent in normal cells (as demonstrated in our RPE study), while at higher concentrations, it can selectively induce apoptosis in cancer cells, making it a promising antitumoral agent [52]. This selective cytotoxicity has been extensively documented in cancer research, where curcumin concentrations that are cytotoxic to cancer cells (typically 10–50 μM) often spare normal cells, highlighting its therapeutic potential in oncology. For instance, studies have shown that low concentrations of curcumin may protect hepatocytes by reducing lipid peroxidation, while higher concentrations provoke apoptosis specifically in cancer cells, with minimal effects on normal hepatocytes [53]. This concentration-dependent selectivity between normal and malignant cells represents a fundamental advantage of curcumin’s hormetic profile and explains its dual role as both a cytoprotective and antitumoral agent. This hormetic dose–response relationship is particularly relevant for polyphenolic compounds like curcumin, which can function as both antioxidant and pro-oxidant depending on concentration and cellular context. This aligns with our previous work [32] and underscores the critical importance of dosage optimization in therapeutic applications. The narrow therapeutic window observed—where just a two-fold increase from 10 μM to 20 μM results in cytotoxicity—is characteristic of hormetic compounds and reflects the transition from curcumin’s antioxidant to pro-oxidant properties. At concentrations ≥ 20 μM, curcumin likely generates excessive reactive oxygen species, disrupts mitochondrial membrane potential, and engages in non-specific protein binding, leading to cellular toxicity [51].

One of the most striking findings of this study is the demonstration that curcumin’s protective effects in RPE cells may be mediated through SIRT1 enzymatic activity inhibition, rather than through its activation. This challenges the conventional view that SIRT1 activation is universally beneficial for cellular protection [54,55]. Our data reveal that moderate oxidative stress induced by H_2_O_2_ caused a significant decrease in acetylated p53 levels, indicating SIRT1 activation, which was associated with increased cellular damage. Importantly, curcumin treatment at 10 μM restored acetylated p53 levels, suggesting an inhibition of SIRT1 activity.

The paradoxical activity of SIRT1 in RPE cells is further supported by our experiments with selective SIRT1 modulators. SRT1720, a SIRT1 activator, showed dose-dependent cytotoxicity and failed to protect cells against oxidative stress. Conversely, the SIRT1 inhibitor EX527 at 20 μM significantly improved cell viability under oxidative stress conditions. These findings suggest that in the context of RPE cells under oxidative stress, SIRT1 hyperactivation may be detrimental rather than protective. This apparent contradiction with the established literature can be explained by the concept of biphasic and dose-dependent action and cellular context-dependency. While SIRT1 activation is generally considered beneficial for longevity and stress resistance [56], excessive activation may lead to cellular dysfunction [57]. In RPE cells, which are already metabolically active and exposed to continuous oxidative stress due to their role in photoreceptor support and phagocytosis, additional SIRT1 activation may push cells beyond their adaptive capacity.

The modulation of p53 acetylation represents a key mechanistic insight provided by our study. SIRT1-mediated deacetylation of p53 at lysine 382 typically leads to reduced p53 transcriptional activity and decreased expression of pro-apoptotic genes. However, in the context of oxidative stress in RPE cells, p53 deacetylation appears to be associated with increased vulnerability to cell death. Our findings suggest that maintaining p53 in its acetylated, transcriptionally active state may be crucial for RPE cell survival under stress conditions. This observation aligns with emerging evidence that p53 acetylation status determines the cellular fate in response to stress [58]. While deacetylated p53 may promote cell cycle arrest and DNA repair under mild stress conditions, acetylated p53 may be necessary for implementing protective responses under severe oxidative stress, including the activation of antioxidant response pathways and DNA repair mechanisms.

Our study also demonstrates that curcumin’s protective effects extend beyond SIRT1 pathway modulation to include direct anti-apoptotic mechanisms. Both curcumin concentrations, 5 and 10 μM, significantly reduced cleaved caspase-3 levels and its translocation into the nucleus, indicating inhibition of the apoptosis process. This finding is consistent with curcumin’s well-established anti-apoptotic properties and suggests that its protective effects in RPE cells involve multiple complementary mechanisms [59].

The ability of curcumin to inhibit caspase-3 activation was observed at both tested concentrations, while only the higher concentration (10 μM) effectively modulated SIRT1 activity, suggesting that these mechanisms operate independently and may have different curcumin concentration thresholds. This differential concentration-dependency further supports the hormetic nature of curcumin’s actions, where different biological pathways respond to distinct concentration ranges within the therapeutic window. The anti-apoptotic effects occurring at lower concentrations (5 μM) may represent the most sensitive hormetic response, while SIRT1 modulation requires higher concentrations (10 μM) within the beneficial range. This concentration gradient effect is consistent with curcumin’s well-established dose-dependent selectivity: the same concentrations that provide cytoprotection to normal cells like RPE can simultaneously exert antitumoral effects in malignant cells, suggesting that cancer cells have enhanced sensitivity to curcumin-induced apoptosis compared to normal cells [52]. This selective vulnerability of cancer cells to curcumin’s pro-apoptotic effects, even at concentrations that are protective for normal cells, underscores the therapeutic potential of exploiting curcumin’s hormetic properties for both neuroprotection and cancer treatment. This multi-target approach may explain curcumin’s robust protective effects and its potential advantages over single-target therapeutic approaches in AMD.

The findings of this study have significant implications for AMD treatment strategies. Current therapeutic approaches for AMD are limited [60], and the identification of curcumin’s protective mechanisms provides a foundation for developing new interventions. The dual action of curcumin—inhibiting excessive SIRT1 activation while preventing apoptosis—suggests that it may be particularly effective in early AMD stages when RPE dysfunction is beginning but cell death has not yet become extensive. The hormetic properties of curcumin present both opportunities and challenges for therapeutic development. On one hand, the ability to achieve protective effects at relatively low concentrations may minimize side effects and improve safety profiles. On the other hand, the narrow therapeutic window and steep dose–response curve necessitate precise dosing strategies to maintain concentrations within the beneficial range while avoiding the pro-oxidant effects that occur at higher concentrations.

The biphasic response to curcumin concentrations suggests that the protective effect will require precise dosing to achieve benefits while avoiding toxicity. This challenge is further complicated by curcumin’s poor bioavailability and rapid metabolism [61], which may necessitate the development of novel delivery systems such as topical administration.

Understanding the hormetic nature of curcumin’s effects will be crucial for optimizing delivery systems and dosing regimens, as maintaining tissue concentrations within the narrow therapeutic window will be essential for clinical efficacy. While our study provides valuable insights into curcumin’s protective mechanisms, several limitations should be acknowledged. First, the use of a single cell line (ARPE-19) may not fully recapitulate the complexity of primary RPE cells or the in vivo environment. Future studies should validate these findings in primary RPE cells and AMD animal models. Second, the acute oxidative stress model used in this study may not fully represent the chronic, low-level oxidative stress characteristic of AMD pathogenesis. Third, the hormetic effects observed in vitro may be influenced by factors such as cell culture conditions, passage number, and experimental duration, which could affect the concentration thresholds for beneficial versus harmful effects. Future studies should systematically evaluate these variables to establish robust dose–response relationships that can inform clinical translation.

In conclusion, this study provides novel evidence that curcumin protects RPE cells from oxidative stress-induced damage through mechanisms that include SIRT1 pathway modulation and caspase-3 inhibition. Importantly, our findings suggest that in the context of RPE cells under oxidative stress, SIRT1 inhibition rather than activation may be protective. The demonstration of curcumin’s hormetic dose–response profile adds a critical dimension to our understanding of its therapeutic potential, emphasizing that the concentration-dependent nature of its effects must be carefully considered in any clinical application.

This research highlights the importance of understanding tissue-specific and context-dependent effects of SIRT1 modulation, particularly in retinal cells, where oxidative stress plays a crucial role in pathology. Moreover, the recognition of curcumin’s hormetic properties provides a framework for understanding why previous studies may have reported conflicting results, as the beneficial or harmful effects likely depend on the specific concentrations tested and cellular contexts employed. The dual nature of curcumin as both a cytoprotective agent for normal cells and a cytotoxic agent for cancer cells at overlapping concentration ranges represents a unique therapeutic advantage. Indeed, this selectivity suggests that curcumin’s mechanism of action may be strictly dependent on the metabolic conditions of the cells and their extracellular environment.

Furthermore, the identification of dual protective mechanisms of curcumin provides a foundation for developing more effective treatments for AMD and other retinal degenerative diseases, while emphasizing the critical importance of dose optimization in therapeutic applications. Future therapeutic development should incorporate the principles of hormesis to optimize curcumin’s beneficial effects while minimizing potential adverse outcomes, potentially through the development of sustained-release formulations or combination therapies that maintain optimal tissue concentrations within the protective dose range.

## 4. Materials and Methods

### 4.1. Cell Culture

The experiments were carried out on the human retinal epithelium ARPE-19 cell line, purchased from the American Type Culture Collection (ATCC, Manassas, VA, USA). Cells were cultured in a medium containing a 1:1 mixture of Dulbecco’s Modified Eagle Medium (DMEM) and Ham’s F-12 (DMEM/Ham’s F-12 w/o L-Glutamine with 15 mM HEPES–Sial, Rome, Italy) supplemented with 10% Fetal Bovine Serum (FBS) (Sial, Rome, Italy), 1% glutamine, and 1% penicillin/streptomycin (Euroclone, Milan, Italy). Cells were cultured in a humidified incubator at 37 °C with 5% CO_2_ and used between passages 6 and 20.

### 4.2. Curcumin Treatment and Oxidative Stress Induction

Curcumin (Cur) powder was resuspended in DMSO (Merk Life Science, Milano, Italy, Cat. Num. D8418) to obtain a 10 mM stock solution. Five thousand cells/well of ARPE-19 were plated and grown for 24 h in ninety-six-well plates (Corning, Turin, Italy). Curcumin up to 50 µM is non-cytotoxic [34], and, to determine the best lower curcumin concentration to use in our experiments, we tested 5, 10, 20, 50, and 100 µM curcumin concentrations for 24 h. Subsequently, in order to establish the right time of curcumin treatment, we performed experiments setting up 3 different time points (4-8-24 h) as reported in (Figure S1) with the selected curcumin concentrations 5 and 10 µM. Furthermore, the cells were treated with increasing concentrations of H_2_O_2_ (200, 400, 600, 700, 800, and 1000 µM) to select the best concentration able to decrease cell viability in a range between 80 and 50%. Based on this pilot experiment, we designed the experimental procedure as follows: 24 h of curcumin treatment followed by 600 or 700 µM of H_2_O_2_ for 24 h. MTT assay (Thiazolyl Blue Tetrazolium Bromide, Merck, Milan, Italy, Cat. Num. M2128) was performed to evaluate cell viability in all experimental groups.

### 4.3. SIRT1 Activator (SRT1720) and Inhibitor (EX527) Administration Under Physiological and Oxidative Stress Conditions

ARPE-19 cells were treated with SRT1720 (Merck, Milan, Italy, Cat. Num. 567860) (1 µM, 2.5 µM, 5 µM, 7.5 µM, 10 µM, 15 µM, and 20 µM), a selective SIRT1 activator, or its inhibitor EX527 (Sigma Aldrich, Cat. Num. E7034) (1 µM, 2.5 µM, 5 µM, 7.5 µM, 10 µM). SRT1720 and EX527 powders were dissolved in DMSO to obtain 5 mM and 80 mM stock solutions, respectively. Cell viability assay was performed to determine the cytotoxic concentrations of both the activator and the inhibitor. To evaluate any potential harmful effects of the vehicle on cell viability, cells were treated with DMSO [0.2%]. Non-cytotoxic concentrations of SRT1720 (1 µM, 2.5 µM, and 5 µM) and EX527 (1 µM and 20 µM) were used to evaluate their effect on ARPE-19 cells exposed to H_2_O_2_ through MTT assay, performed according to the manufacturer’s instructions as described in the next paragraph.

### 4.4. Cell Viability Assay

First, 10 µL of MTT solution, diluted 1:10 to a final volume of 100 µL (5 mg/mL), was added to each well. The cells were incubated in a humidified atmosphere at 37 °C and 5% CO_2_ for 2 h. After incubation, the MTT solution was removed, and 100 µL of DMSO was added to dissolve the formazan crystals produced by the viable cells. At the end of the procedure, the absorbance was measured at 570 nm using a microplate reader (Tecan Infinite^®^ 200 PRO, Tecan, Männedorf, Switzerland).

### 4.5. Protein Extraction and Western Blot Analysis

Western Blot analysis was performed to quantify SIRT1, acetyl-p53, and caspase-3. ARPE-19 cells were plated in 10 cm Petri dishes (Corning, Turin, Italy) at a density of 650,000 cells and incubated for 24 h at 37 °C with 5% CO_2_ and then treated with curcumin (5 µM or 10 µM) and H_2_O_2_ (700 µM), according to the experimental setup. After the treatments, the medium was removed, and the cells were gently washed with 5 mL of PBS 1X (Merck, Milan, Italy). Then, the cells were harvested using 1 mL of PBS 1X and centrifuged at 4000 rpm for 6 min at 4 °C, and the supernatant was discarded. Protein extraction was then performed using a lysis buffer (50 mM TrisHCl, pH 7.5, 1% Triton X-100, 0.1% SDS, 5 mM EDTA, Thermo Fisher Scientific Protease and Phosphatase Inhibitor Cocktail, and QS dH_2_O). The cell suspension was kept on ice for 30 min, and cells were vortexed every 10 min to promote efficient protein extraction. Next, the lysate was centrifuged at 13.200 rpm for 20 min at 4 °C, and the supernatant was collected. The protein concentration was determined by BCA assay (Thermo Fisher Scientific, Waltham, MA, USA, Cat. Num. B14). Then, 40 µg of the protein lysates was resolved by SDS-Polyacrylamide gel electrophoresis (PAGE), transferred onto PVDF membranes (BioRad Laboratories, Hercules, CA, USA), and placed in 5% Bovine Albumin Serum (BSA) or non-fat dry milk in TBST buffer (25 mM Tris-HCl pH 8.0, 125 mM NaCl, 0.1% Tween 20) to block non-specific binding to the membrane. The membranes were then incubated with the indicated primary antibodies: anti-SIRT1 (05-1243 Millipore, 1:1000); anti-acetyl-p53 (Cat. Num. 2525S Cell Signaling, Leiden, The Netherlands), 1:500; anti-caspase-3 (Cat. Num. 9662, Cell Signaling, Leiden, The Netherlands 1:1000); anti-GAPDH (Cat. Num. 2118 Cell Signaling, 1:10,000). Secondary horseradish peroxidase-conjugated goat anti-rabbit or goat anti-mouse antibodies (BioRad Laboratories, Hercules, CA, USA) were used at a 1:5000 dilution; the protein bands were visualized by enhanced chemiluminescence (ECL) (BioRad Laboratories, Hercules, CA, USA) using the Chemidoc MP imaging system (BioRad Laboratories, Hercules, CA, USA).

### 4.6. Immunofluorescence

The ARPE-19 cells at a density of 5 × 10^4^ cells/well were seeded on a 12 mm coverslip (Merck, Milan, Italy) sterilized with ethanol 100% (Sial, Rome, Italy), placed in 24-well plates (Corning, Turin, Italy) coated with 1.5% gelatine for 5 min and grown until confluence (at 37 °C in 5% of CO_2_). The cells were fixed with PAF 4% for 10 min. The non-specific binding sites were blocked with BSA 3% in PBS-T 0.1% for 1 h at room temperature (RT). The primary antibody anti-caspase-3 (1:1000, Cell Signaling, Cat. Num. 9662) was incubated in a BSA1%-PBS solution overnight at 4 °C. The following day, after 3 PBS 1x washes, the cells were incubated with the secondary antibody (Alexa fluor goat anti-rabbit 594, Thermo Fisher Scientific, Waltham, MA, USA, Cat. Num. A11012) for 1 h at 37 °C. Negative control was only incubated with the secondary antibody. The nuclei were counterstained with Bisbenzimide (Hoechst 1:50.000, Merck, Milan, Italy, Cat. Num. 14533) for 2 min, and the samples were mounted with Glycerol Gelatine (Merck, Milan, Italy, Cat. Num. GG1). Confocal Images were acquired through a Leica Stellaris 8 TauSTED (Leica Microsystems, Milan, Italy).

### 4.7. Statistical Analysis

All experiments were performed in biological triplicates, each with at least one technical replicate. Data are presented as Mean ± Standard Error (SE), and statistical analyses were conducted using Sigma Plot 15.0 software. The statistical test used was One-Way ANOVA followed by Holm–Sidak Test, Tukey, or SNK test for the comparison of two or more experimental groups. The first type error was set at 5%.

## Data Availability

All data supporting the findings of this study are available by contacting the corresponding author, R.M.

## Supplementary Materials

The following supporting information can be downloaded at https://www.mdpi.com/article/10.3390/ijms26178555/s1.

## Abbreviations

The following abbreviations are used in this manuscript:

RPE	Retinal Pigment Epithelium
AMD	Age-related Macular Degeneration
ROS	Reactive Oxygen Species
BRB	Blood–Retinal Barrier
SIRT1	Sirtuin 1
HDACs	Histone Deacetylases
CDK	Cyclin-Dependent Kinase
NF-kB	Nuclear Factor kappa-light-chain-enhancer of activated B cell
ATCC	American Type Culture Collection
DMEM	Dulbecco’s Modified Eagle Medium
FBS	Fetal Bovine Serum
CUR	Curcumin
MTT	Thiazolyl Blue Tetrazolium Bromide
PAGE	SDS-Polyacrylamide gel electrophoresis
BSA	Bovine Serum Albumin
RT	Room Temperature
SE	Standard Error
