# Supplementary material for: Hormetic Effects of Curcumin in RPE Cells: SIRT1 and Caspase-3 Inactivation with Implications for AMD
Source: Int J Mol Sci. 2025 Sep 3;26(17):8555. doi: 10.3390/ijms26178555 (PMC12429762; doi:10.3390/ijms26178555)
Supplement: Supplementary file 1 [file ijms-26-08555-s001.zip › ijms-3792034-supplementary.docx]

**SUPPLEMENTARY FIGURE 1**

**
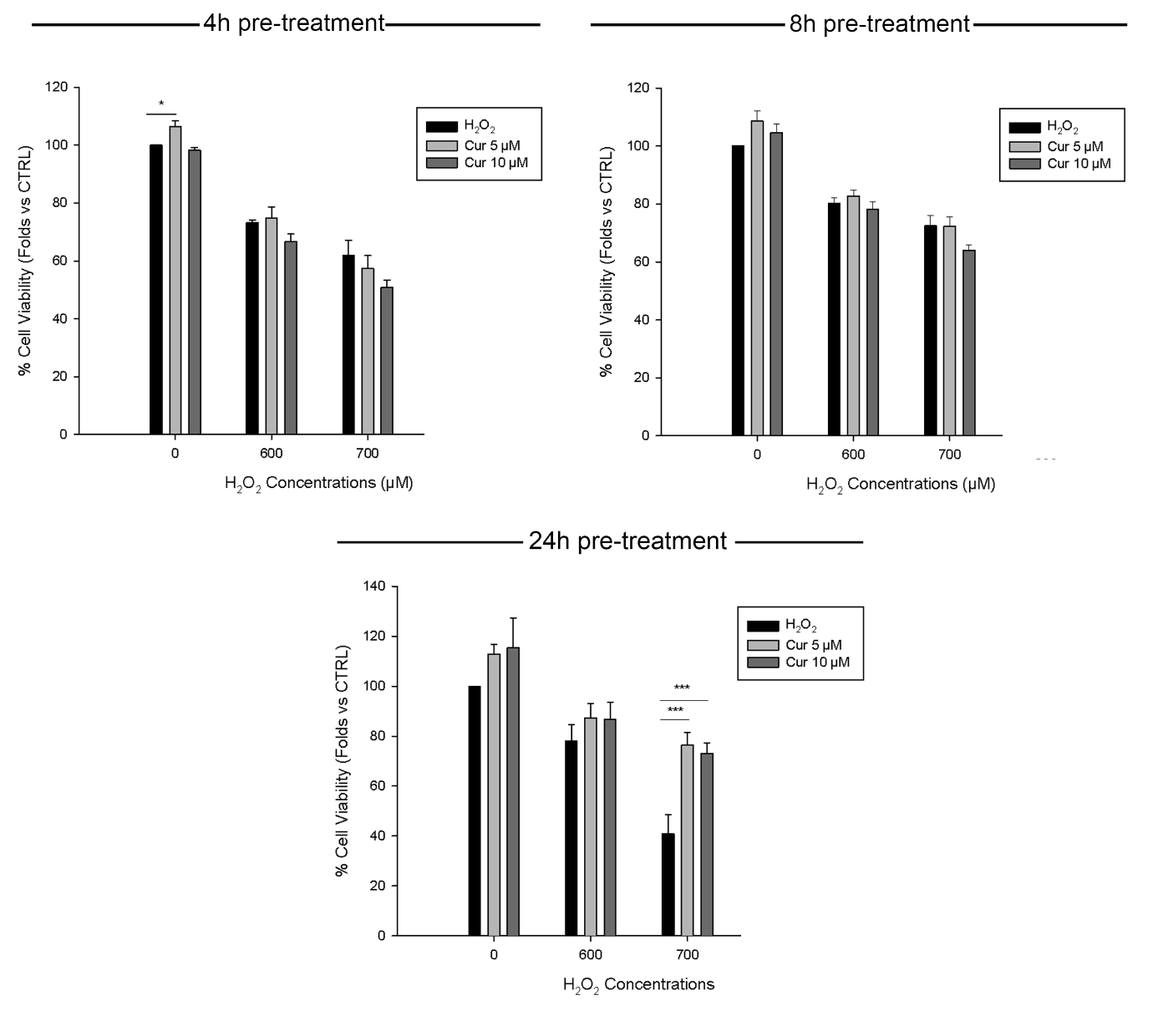
**

**Supplementary Figure 1.** (A) MTT assay to evaluate cell viability in ARPE-19 cells pre-treated with curcumin (5 and 10 µM) for 4 hours, followed by treatment with H₂O₂ (600 µM and 700 µM). (B) MTT assay to evaluate cell viability in ARPE-19 cells pre-treated with curcumin (5 and 10 µM) for 8 hours, followed by treatment with H₂O₂ (600 µM and 700 µM). (C) MTT assay to evaluate cell viability in ARPE-19 cells pre-treated with curcumin (5 and 10 µM) for 24 hours, followed by treatment with H₂O₂ (600 µM and 700 µM). Abbreviation: CTRL; control and Cur; curcumin. Statistical analysis was performed using One-Way ANOVA followed by Holm-Sidak Test (n=3-6). * p < 0.05; *** p < 0.001. Data are shown as Mean ± SEM.
